# Signal honesty and predation risk among a closely related group of aposematic species

**DOI:** 10.1038/srep11021

**Published:** 2015-06-05

**Authors:** Lina María Arenas, Dominic Walter, Martin Stevens

**Affiliations:** 1 Department of Zoology, University of Cambridge, Downing Street, CB2 3EJ, UK; 2 Centre for Ecology & Conservation, College of Life & Environmental Sciences, University of Exeter, Penryn Campus, Penryn, Cornwall, TR10 9FE, UK

## Abstract

Many animals have bright colours to warn predators that they have defences and are not worth attacking. However, it remains unclear whether the strength of warning colours reliably indicate levels of defence. Few studies have unambiguously established if warning signals are honest, and have rarely considered predator vision or conspicuousness against the background. Importantly, little data exists either on how differences in signal strength translate into survival advantages. Ladybirds exhibit impressive variation in coloration both among and within species. Here we demonstrate that different levels of toxicity exist among and within ladybird species, and that signal contrast against the background is a good predictor of toxicity, showing that the colours are honest signals. Furthermore, field experiments with ladybird models created with regards to predator vision show that models with lower conspicuousness were attacked more frequently. This provides one of the most comprehensive studies on signal honesty in warning coloration to date.

Aposematic prey use conspicuous or distinctive signals paired with unprofitability to warn predators from attacking, and the strategy is widespread in the animal kingdom^1^,^2^. For example, there is evidence that mammals^3^, birds^4^, amphibians^5^, marine invertebrates^6^ and many insects^7^ use them to deter predators. For warning colours to be effective they need to promote both initial avoidance and aversion learning in predators, and there is some evidence that certain types of colour, and more conspicuous signals, are more effective in achieving this^8^. Recently, an increasing number of theoretical and empirical studies have addressed the issue of whether warning colours should be quantitatively honest indicators of toxicity, whereby the signal provides accurate information about the unprofitability of a potential prey item, with more conspicuous prey being more toxic^9^. However, work so far has often proven contradictory or inconclusive, and conspicuousness has not always been appropriately defined or measured (see below). Thus, considering aposematic signal form in an objective manner, or ideally in terms of observer vision, is of crucial importance to studies on warning signals and animal communication in general^10^.

Recent evidence has shown that there can be variation in the level of toxins among individuals of a species, and that this is related to their conspicuousness. For example, correlations have been found between the size of the poison gland and the conspicuousness of paper wasps (*Polistes dominula*)^11^, and between levels of toxin and elytra coloration in ladybirds^12^. However, most studies use measures of coloration derived from simple measurements from uncalibrated images or do not analyse coloration with regards to a predator’s visual system^13^,^14^ (but see Cortesi & Cheney^6^, Blount *et al.*^9^ and Winters *et al.*^15^). Furthermore, although we may often predict a positive correlation between toxicity and conspicuousness, the actual relationship may sometimes be more complex. For example, both toxicity and coloration may be energetically costly to produce and maintain^16^, and some theoretical models have predicted that once a predator has learned that a prey is toxic, prey should reduce the amount of energy invested in producing an honest signal^17^. This could in turn make aposematism evolutionarily stable, as opposed to a constant arms race between toxicity, coloration and predator perception or toxic resistance^18^. Some evidence in support of these predictions has been found in poison frogs and ladybirds^9^,^19^.

Most work on signal honesty has also focused on within-species variation rather than differences among closely related species. For example, Summers and Clough^5^ measured the coloration (brightness) of poison-arrow frog species (Dendrobatide) and found a positive correlation between toxicity and coloration. However, conspicuousness was analysed using human subjects, who have different visual perception to predators in the wild^14^. Later, Cortesi & Cheney^6^ provided evidence for honesty in the aposematic signals of marine opistobranchs by measuring coloration according to predator visual systems (two species of fish), taking into account the contrast of the signals against the background, and assessing toxicity through bioassays using brine shrimp. While this is an important contribution to the study of honesty in aposematic organisms, the perception of signals in water and terrestrial environments differs considerably^20^, and thus how accurately this finding can be extrapolated to terrestrial systems is unclear. Furthermore, the results of this study are based on laboratory trials, but there is little information of how vulnerable species are to predators in the wild^21^. Moreover, although several studies^22^,^23^ have tested the avoidance of artificial warning coloured models in the wild, these data are not often *directly* correlated to coloration or toxin levels (but see Nokelainen *et al.*^24^). Simply showing that toxicity and appearance are correlated is not enough because this does not mean that predators utilise and respond to measured variation in signal strength in an informed manner, and that this affects prey survival.

While previous studies have found relationships between toxicity and conspicuousness (either direct honesty^11^ or results consistent with resource allocation models^9^), most studies suffer from one of two drawbacks (or both). First, many studies fail to consider predator vision and so it is unclear whether their data on conspicuousness is accurate in terms of how it would be perceived by natural predators^25^. Second, studies frequently fail to consider the conspicuousness of the signals on a natural background against which the prey animal would be found, despite this being an important element determining the perception of warning signals^6^,^8^. Most studies measure conspicuousness as an inherent property of the animal itself with, for example, animals that are more red or yellow being more conspicuousness. However, in most cases conspicuousness is really determined by how visible and contrasting an object is against the background environment where it would naturally occur^2^,^8^. Few studies have analysed conspicuousness against the natural background and compared this to unpalatability^15^,^26^.

Ladybirds (Coloeoptera: Coccinellidae) are a diverse group of beetles that are usually brightly coloured, and it is widely accepted that ladybird colour patterns are important for predator deterrence^9^. In addition, ladybirds have toxic chemicals that are often produced endogenously^9^,^12^,^17^. These chemicals have been found to be correlated with carotenoid abundance in the seven spot ladybird *Coccinella septempunctata*^9^, and the area of background elytra coloration in the Asian ladybird *Harmonia axyridis*^12^. Within the Coccinellidae family, variation in ladybird coloration is impressive, ranging from red, orange and yellow, to brown and nearly white species, and the patterns on the elytra are variable as well^27^. Moreover, many species of ladybird beetles coexist, presumably putting predators in a situation where they have to learn and recognise many colours and patterns and make accurate decisions on whether or not to attack them.

In this study, we aimed to determine whether five species of ladybird beetles (one species having two colour morphs) that have some of the most common warning colours (namely, red, orange, yellow and black^1^,^2^) advertise their toxicity honestly among and within species. We measured coloration using digital image analysis and avian visual modelling to make accurate predictions about conspicuousness^14^. Also, we measured the strength of the chemical defense of each ladybird species using toxicity bioassays with water fleas (*Daphnia pulex*; see Methods). Although we are aware of the value of using real predators (when we can be confident of their ecological relevance) in experimental studies on aposematism, our bioassays provide and objective method that yields results independent of any potential coevolution between predator resistance and prey toxicity levels^6^. For example, if predators have evolved resistance to certain prey defenses then this can create difficulties in objectively assessing prey toxicity. This may be especially problematic if the predator species used has differing levels of evolved resistance towards different prey species tested, especially if the predator targets some species more than others (which is possible when potential prey species occupy different niches). Our method here circumvents this issue with an objective approach. Note that we consider both bioassays and experiments with real predators (when we can be confident that they are ecologically relevant) are valuable in studying aposematism. Additionally, although we were only working with five species, we calculated two different estimators of phylogenetic signal to determine if our correlations between coloration and toxicity could be influenced by the relatedness of the species. These analyses showed no evidence for a phylogenetic signal, although are somewhat inconclusive when dealing with small sample sizes^28^ (see supplementary material S1). Finally, we measured ladybird predation rates in the field using artificial models of aposematic and non-aposematic ladybirds. In this study we only consider visual cues and the initial avoidance of predators. Thus, our models were calibrated to predator (avian) vision, and were all equally palatable and of the same size, enabling us to determine whether differences in survival relate to coloration alone. We also measured the conspicuousness of the models against the natural backgrounds where they were placed to determine how conspicuousness affects predation risk. Our overall aim was to provide a comprehensive study of the function of warning signals among a group of closely related aposematic species and test whether warning signals in these beetles are quantitatively and qualitatively honest^17^. Furthermore we aimed to establish if bird predators rely on specific colour cues to avoid certain species of ladybirds, or if they effectively generalise their predatory decisions based on a broad rejection rule to avoid prey with more conspicuous coloration^7^.

## Results

### Toxicity analysis

**Figure 1:**
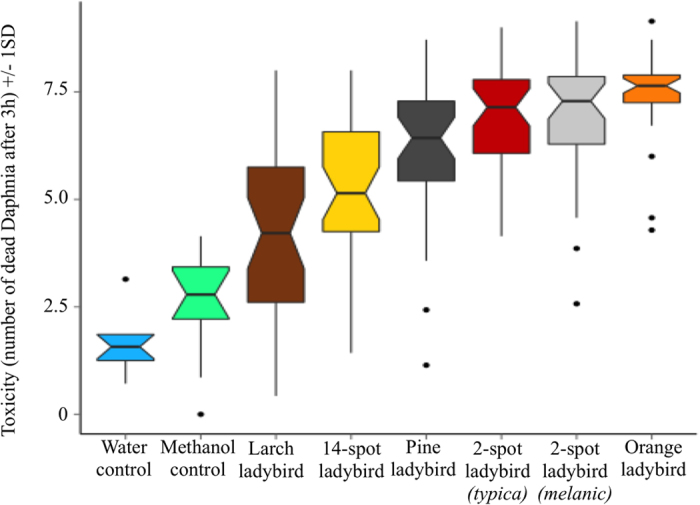
Toxicity of the different ladybird species analysed in this study. There were significant differences between all species tested, while controlling for the effects of size and weight. Both the water and the Methanol controls were less lethal to the *Daphnia* individuals than any of the toxin extracts analysed. Larch ladybirds are the least toxic species followed by the yellow 14-spot ladybirds. Pine ladybirds are more toxic than those previous two species. The two morphs of 2-spot ladybirds are indistinguishable in their toxicity, but less toxic than the orange ladybird, which has the highest toxicity. The notches on each boxplot represent the 95% confidence interval for each species.

We measured ladybird toxicity using bioassays on *Daphnia pulex* with ladybird toxin extracts that were diluted into seven concentrations. We found that the ladybird species used for this study have different toxicities (CoxME, DF = 49; X2 = 2774.9; p < 0.001; LME, DF = 5, 54; F = 20.209; lower p < 0.001). As Fig. 1 shows, the orange ladybirds were the most toxic species, followed by the 2-spot ladybird, which showed no differences between the two morphs analysed. The third most toxic species was the pine ladybird, with 14-spot ladybirds being the species with aposematic coloration that had the lowest toxicity. Larch ladybirds had the lowest toxicity of all the species analysed. These general trends were influenced by the dilution of the toxin extracts (CoxME (species*dilution, DF = 42 = X^2^ = 352.5, p < 0.001, see Supplementary material S2 for all pairwise comparisons in each toxin dilution). In addition, although the Methanol control was more toxic to the *Daphnia* than water, both this and the water control were significantly less toxic than any of the ladybird extracts.

### Signal honesty

**Figure 2:**
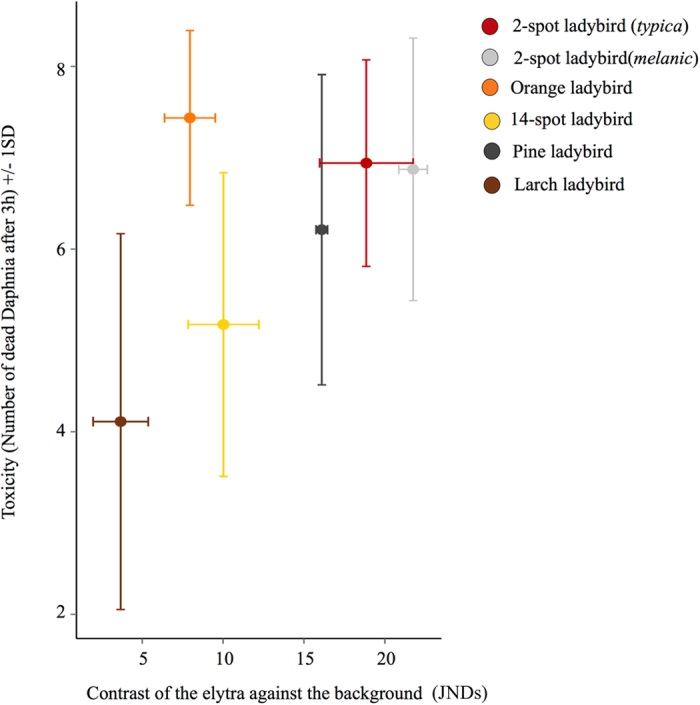
Contrast against the species’ preferred background in terms of colour contrast (just noticeable differences; JNDs +/− SD) and its relation to toxicity. Species with higher toxicity are more contrasting than less toxic species. The only exception is the orange ladybird, which despite being the most toxic species is of intermediate conspicuous.

**Figure 3:**
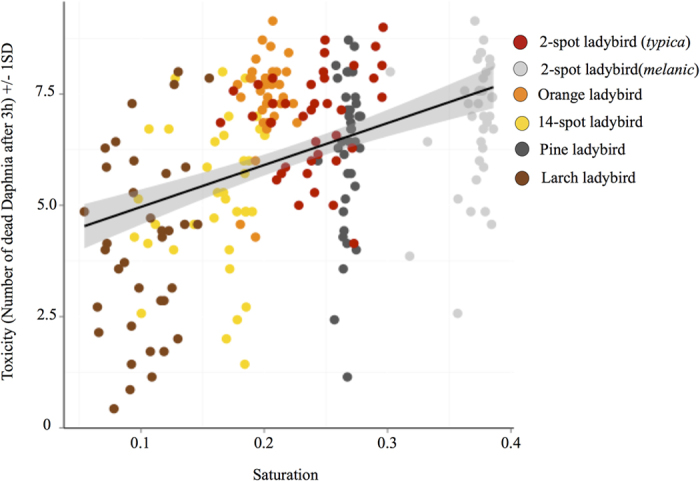
Correlation between saturation and toxicity within species, the more saturated individuals in each species are more toxic. These results are true for all species except the orange ladybird. The shaded grey area shows the standard deviation of the general trend line.

We used digital image analysis and vision modelling to test if ladybird beetles advertise their level of toxicity with honest signals. We quantified this with measures of overall elytra luminance (double cone values reflecting the perceived lightness of the animal to an avian predator^29^) and saturation (colour richness; i.e. pink versus red). In addition, we quantified the area of background colour of each ladybird’s elytra, as well as both the colour and luminance contrast against the background where each species is normally found, and the internal luminance contrast (i.e. elytra background against spots), and determined whether the above measures were effective as predictive variables of toxicity. Of the five signal attributes we correlated with toxicity (log of the average number of dead *Daphnia* after 3 hours), we found that toxicity is predicted by the colour contrast measured in terms of predator discrimination thresholds (‘just noticeable differences’ or JNDs) against the background for each species (LME, Species*Contrast (own); DF = 5, 54; F = 2.786; lower p = 0.026; Fig. 2), but not luminance contrast against the background (LME, DF = 5, 37; F = 0.29; lower p = 0.592). Figure 2 shows that the species with high toxicity have higher contrast, except for the orange ladybird. In addition, we found that the most toxic individuals *within* each species have also more saturated elytra (LME, DF = 1, 54; F = 5.57; lower p < 0.05; Fig. 3), and we found a correlation between saturation and contrast, whereby more saturated individuals within each species are also more contrasting (LME, Saturation*Contrast (own); DF = 1, 54; F = 4.338; lower p = 0.042). Finally, we found that internal luminance contrast between spots and elytra does not predict the toxicity of any of the species analysed (LME, DF = 5, 37; F = 0.31; lower p = 0.580; Supplementary material S3.

To test the possibility that the Larch ladybird alone could drive some of our results owing to its lack of toxins and non-aposematic coloration, we reran all of the analyses above excluding this species. This model shows that species remains a good predictor of toxicity (LME, DF = 4, 44; F = 14.29; lower p < 0.001). In addition, interspecific correlations between toxicity and contrast against the background (LME, DF = 4, 44; F = 3.25; lower p < 0.05), as well as, intraspecific correlations with saturation (LME, DF = 1, 44; F = 5.70; lower p < 0.05) remain, even when excluding the Larch ladybird.

### Predation in the field against wild predators

**Figure 4:**
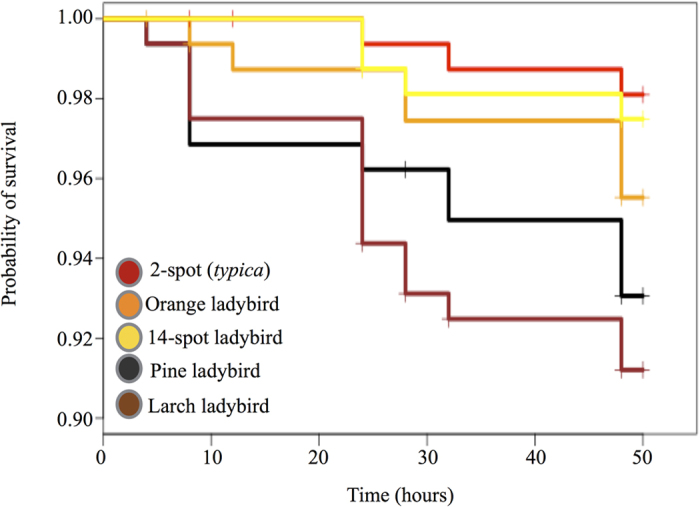
Survival probability in wild environments for each of the five colours of models used in this experiment. Red, orange, and yellow models have higher survival rates than brown and black models. The average predation time across all models and all transects was 28 hours.

**Figure 5:**
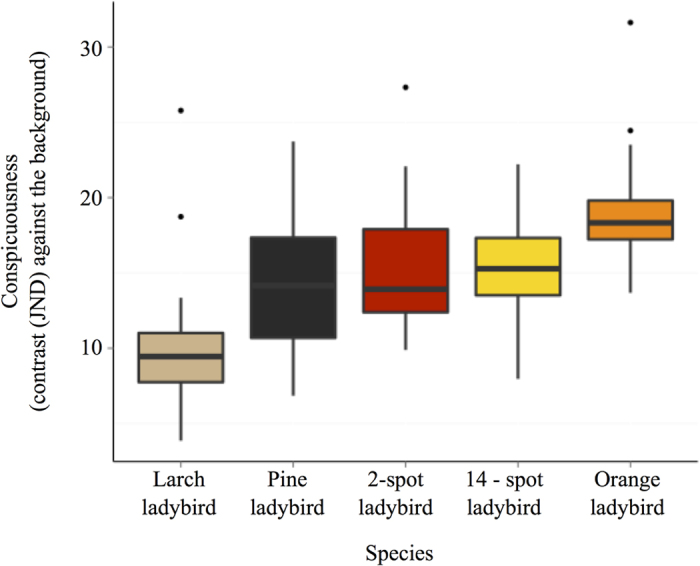
Conspicuousness of the artificial ladybird models against natural backgrounds. Brown models are the least conspicuous, while bright colours such as orange and yellow are highly contrasting. The red and black models showed no differences in their contrast despite their different survival rates.

We built artificial ladybirds, modelled to avian vision to quantify predation rates in the field. Using an acrylic mould, we manipulated colour-printed paper of different colours to a ladybird shape. The models were pinned on leaves and checked over 48 hours to record any predation events. Of the 750 artificial models we placed along 15 transects, 50 (6%) were attacked. This attack rate is the same or even higher than many other experiments involving model aposematic prey e.g. Merrill *et al.* (2012)^23^, whereby we expect predators to have learnt and innate avoidance of such colours. Even with relatively low levels of predation, our results showed that there are differences in the survival of the models used in this study (CoxME (species, DF = 1= X^2^ = 3.0682, p < 0.05; Fig. 4). We found that the red models (representing 2-spot ladybirds), orange models (corresponding to orange ladybirds), and yellow models (representing 14-spot ladybirds) were not different in their survival (red vs. orange: coef = −0.86; z = −1.26; p = 0.21; orange vs. yellow: coef = −0.58; z = −0.93; p = 0.35; red vs. yellow: coef = 0.28; z = 0.37; p = 0.71). However, the brown (representing larch ladybirds) models were attacked more than the red (coef = 1.58; z = 2.49; p < 0.05), and more than the yellow models (coef = 1.29; z = 2.29; p < 0.05). In addition, the black (imitating pine ladybirds) models did not survive differently from the brown models (coef = 0.25; z = 0.63; p = 0.53), but were less likely to survive than the red (coef = 1.32; z = 2.04; p < 0.05) and the yellow models (coef = −1.04; z = −1.79; p < 0.05). Importantly, we found that the models with lower colour contrast are attacked in higher proportions (LME Species*Colour Contrast; DF = 4, 137; F = 4.5645; lower p < 0.001; Fig. 5). The brown models survive less well and have lower conspicuousness than the black (DF = 4, 137; t = −2.433; p < 0.005), orange (DF = 5, 137; t = −6.028; p < 0.001), and yellow (DF = 4, 137; t = −4.422; p < 0.001) models. The black models have a lower probability of survival and are less conspicuous than the orange (DF = 4, 137; t = 9.441; p < 0.001) and yellow models (DF = 4, 137; t = 6.255; p < 0.001). However, their conspicuousness (colour contrast) is not different from the red models (DF = 4, 137; t = −1.244; p = 9.01).

## Discussion

Our results show that a closely related group of highly variable species differ in their level of toxicity, and with the exception of one species (the orange ladybird), this correlates with their level of conspicuousness against their natural backgrounds. Furthermore, we found that the more contrasting species also have more saturated colours, and these two variables are significant predictors of toxicity. We did not, however, find a correlation between luminance and toxicity. Unlike Cortesi & Cheney^6^ we found no evidence that the internal contrast of the ladybirds was correlated to their chemical defences. Importantly, we were able to establish that models resembling the different ladybird species to predator vision show differences in their survival rates consistent with their level of conspicuousness (colour contrast) against the background. Our study shows that ladybird aposematic signals are generally honest, both among and within species, for both the elytra coloration and perhaps more importantly their conspicuousness against natural backgrounds. Furthermore, we have demonstrated that wild avian predators respond to levels of conspicuousness, leading to differences in survival. Ours is one of the first studies to comprehensively demonstrate honesty in warning signals both in terms of predator vision and conspicuousness against the background, and the first to show empirically that this translates directly into a difference in survival probability^24^.

In our study, orange ladybirds (*Halyzia sedecimgutata*) were the most toxic species, but not the most contrasting. This may be explained by how life history adaptations influence a species’ survival in its natural environment. For example, orange ladybirds are commonly found in deciduous forests, usually associated with sycamore trees (*Acer pseudoplatanus*)^30^. Interestingly, individuals are normally found basking and/or feeding on the underside of the leaves (LMA, *pers. obs*), indicating that they might be relying on the plant to avoid detection. As a result, orange ladybirds may not need to invest in a particularly conspicuous signal since the probability of being seen from below is lower than from above (e.g. a bird in flight foraging for food). When searching from above a predator may observe the silhouette of the ladybird through the leaf, meaning that the signal would change. Likewise, in the case of pine ladybirds (*Exochomus quadripustulatus*), their small size is often enough to conceal them, at least from human observers (LMA, *pers. obs*). In addition to their aposematic colours^31^, yellow ladybirds (*Propylea quattuordecimpunctata*) may increase their survival through a fast-dropping reflex as a predator approaches (LMA, *pers. obs*). This behaviour has been described for other ladybirds and aphids as a mechanism of protection against predation^27^. Therefore, life history and behavioural traits may also affect the relationship between toxicity and signal form/conspicuousness, and this is an area where future work would be valuable.

Our results, along with the work by Cortesi & Cheney (2010)^6^ provide evidence for the existence of signal honesty among species, and confirm other previous findings^9^,^12^,^15^ supporting a correlation between toxicity and coloration within species. However, in addition to the above, we have also shown how the differences in signal honesty and conspicuousness translate into a survival advantage in a field setting with wild predators. Our results also demonstrate that when measuring conspicuousness it is crucial to measure how individuals contrast with their natural backgrounds, and that much of conspicuousness may be explained by this property, rather than limiting analyses of coloration to the inherent appearance (e.g. saturation and brightness) of individuals. While there can be a correlation between traits such as saturation and toxicity within species, when comparing these features among species, this relationship may not hold. Furthermore, the survival rates of our models in the field illustrates that avian predators seem to base their attack decisions on general rules of conspicuousness rather than an assessment of every potential prey item they encounter. This may not come as a surprising result since generalization has been shown to occur in several species e.g. Ham *et al.* (2006)^32^. These studies suggest that generalization is more effective after negative experiences. In addition, Nokelainen *et al.*^33^ and Mappes *et al.*^21^ have recently shown that predator communities influence the prevalence of warning coloured prey. This suggests that fledglings attack more aposematic prey earlier in the year than after gaining a few months of foraging experience, benefiting the prey later in the season^32^. However, unlike the studies above, our results take into account coloration as seen from a predator’s perspective into the design of the prey items.

Our results are also consistent with work on other forms of protective coloration^34^. For example, many lepidopterans and other insect orders have concentric circular patterns often called ‘eyespots’^35^. These markings often incorporate contrasting colours that may serve as deterrent signals to predators. Such eyespots are also often revealed when the animal is disturbed, startling the predator^36^. Most accounts suggest that eyespots mimic predator eyes, but there is evidence that the effectiveness of eyespot markings can often be explained by features such as contrast between the marking components and the rest of the animal. Furthermore, the benefit of eyespots to survival can switch from being detrimental on otherwise camouflaged prey (presumably through increased detection risk) to being beneficial in deterring predators when on prey that are already conspicuous against the background^35^. Aside from anti-predator coloration, high contrast and conspicuousness against the background environment has also shown to be important in tasks such as mate choice, for example in birds^37^. As such, contrast and conspicuousness of signals, both against the rest of the prey coloration and the wider visual background, are likely to be of widespread importance in signal effectiveness, including aspects relating to honesty.

Beyond immediate aspects of predator-prey interactions other selective pressures can also modulate warning coloration in species. In ladybirds, the colours of 2-spot ladybirds^38^ (among others) can affect mate selection. Sexual selection based on colour patterns has also been demonstrated in *Heliconius* butterflies^23^. In addition, evidence exists regarding trade-offs between conspicuousness and reproductive success. Nokelainen *et al.*^24^ showed that brighter yellow males of the aposematic wood tiger moth (*Parasemia plantaginis*) have lower mating success than white, less toxic males. Moreover, the warning colours of the strawberry poison frog (*Oophaga pumilio*) influence mate choice^39^. The different colour morphs of these species have different toxicity and these may advertise an honest signal for avian predators^40^. In addition to sexual selection, physiology may also influence the effectiveness of an aposematic signal. In several species^41^ including ladybirds^42^ thermoregulatory processes are enhanced by an increment of the melanic pigmentation of body parts. Yet, there is also evidence that the proportion of black coloration can increase predation risk^43^. We have been able to corroborate this in our study by showing that the black models (pine ladybirds) have lower probabilities of survival than species with lower melanisation. Furthermore, an increase in black coloration could decrease the contrast of the aposematic contrast against the background^8^, decreasing the effect of the aposematic signal in time. Thus, further studies that consider aposematism as a multi-modal trait including both prey and predator behaviour should be considered to strengthen our understanding of these issues. Finally, recent evidence has shown that females of the poison frog *Oophaga pumilio* provision their tadpoles with alkaloids via trophic eggs, making their offspring toxic from an early age until they are able to feed on their own^44^. Future studies should also consider the role of inheritance of chemical defences as well as coloration.

It is clear that the honesty of animal signals is an important aspect of communication^45^. Whether signals convey information about a prey’s unprofitability, a mate’s fitness or both, the message that they transmit may determine the evolution of intricate relationships between species. Such is the success of honest signallers, that strategies mimicking coloration patterns have evolved in various species^46^. Further studies on signal honesty should consider multimodal signals, in terms of intra and interspecific interactions and the costs associated to both. This type of integrative studies would allow us to have a more comprehensive view on animal communication systems.

## Methods

### Study species and sites

We included in this study ladybird species that have common aposematic colours. All species have been assessed in terms of their alkaloid contents (but not how toxic these are). The species used were: (1) 14-spot ladybirds (*Propylea quattuordecimpunctata*) which have black spots on yellow elytra and the toxin propyleine^47^ *n* *=* 36, average size = 4.06 mm; (2) Pine ladybirds (*Exochomus quadripustulatus*) which have black elytra and red spots, and the toxin exochomine^48^ *n* *=* 37, average size = 4.05 mm; (3) orange ladybirds (*Halyzia sedecimguttata*) which have white spots on orange elytra. There are no toxins described for this species, but it often reflex bleeds when disturbed, and produces a foul smell (LMA, *pers. obs*) *n* *=* 36, average size = 5.83 mm; (4) 2-spot ladybirds (*Adalia bipunctata*), which have red elytra and black spots and the alkaloids adaline^49^ among others, *n* *= *39, average size = 4.87 mm. We included the black morph of *A. bipunctata* (which has red spots on black elytra), to further test if the different colour morphs of a single species differ in their toxicity, *n* *=* *37,* average size = 4.87 mm; (this black morph was excluded from the survival experiments). (5) We also included the inconspicuous larch ladybird (*Aphidecta obliterata*) as a ladybird species lacking bright coloration and toxins^47^ *n* *=* 36, average size = 4.25 mm. Larch ladybirds were collected at the King’s Forest in Thetford, UK (52°20′17′′N, 0° 39′58′′E). Pine and 14-spot ladybirds were collected in Cambridge, UK (52°12′19.21′′N, 0° 7′18.54′′E), and orange ladybirds were collected in Madingley Woods, Cambridgeshire, UK (52°13′0.98′′N, 0°3′2.93′′E). 2-spot ladybirds were bought from a number of distributors in the United Kingdom. A preliminary test comparing the toxicity and coloration of captive bred (bought) and wild 2-spot ladybirds (*typica*) using the same methods described below, revealed that bought individuals were indistinguishable from wild caught individuals in terms of toxicity (ANOVA, DF = 8, F = 0.513, p = 0.494) and coloration (ANOVA, DF = 1, 0.426, p = 0.515). Supplement S4 shows a diagram of each species represented in a tetrahedral avian vision plot (a) and their mean brightness (b). This plot was created using the LM, MW, SW, and UV values for each of the individuals used in our analyses, and converted to X,Y,Z coordinates using standard methods described by Endler & Mielke (2005)^13^. This graphically displays the different ladybird colours in a three-dimensional space corresponding to avian vision, and has been widely used in studies on coloration e.g. Stoddard & Prum (2008)^50^.

In addition to the ladybirds, we collected samples of the plants where we most commonly found each ladybird species in order to calculate the contrast of each species’ colour against their preferred basking plants. These were: nettle (*Urtica dioica*) for the 2-spot ladybirds and 14-spot ladybirds; sycamore (*Acer pseudoplatanus*) for the orange ladybirds, twigs of Scots pine (*Pinus sylvestris*) for the pine ladybird, and twigs of the European larch (*Larix decidua)* for the larch ladybirds. To obtain an average measurement of the cone catch values of the backgrounds samples, we collected 10 leaves of each plant species, including those where we found the ladybirds. All samples (ladybirds and plant) were obtained during July — August 2013. Ladybirds were euthanized as soon as possible (maximum time being 8 hr) by freezing them in a −80 ˚C freezer for 30 min, to preserve the specimens until the toxicity experiments were conducted. Immediately after euthanizing, the samples were photographed to ensure that there would be no changes in the colour measurements. Previous experiments showed that there is no change in a ladybird’s elytra reflectance within the first 8 hours after collection, using this same euthanizing method (ANOVA, DF = 1; F = 0.358; p = 0.555) (LMA, unpublished). None of the species included in this study are currently endangered or protected by any conservation agency. All experiments were carried out according to local ethical guidelines.

### Image collection and setup

We used a Nikon D90 digital SLR camera to take photographs of the different ladybird species. This camera had undergone a quartz conversion to make its CCD array sensitive to ultraviolet (UV) light (Advanced Camera Services, Norfolk, UK). In addition, the camera was fitted with an AF-S VR Micro-Nikkor 105 mm lens that was sensitive to UV wavelengths. We took photos in the human visible spectrum using a UV/infrared (IR) blocking filter, transmitting between 400–700 nm (Baader UV/IR Cut Filter). In addition, we took comparable ultraviolet photos of each individual using an ultraviolet (UV) pass and IR/visible blocking filter, transmitting between 300–400 nm (Baader U filter). After processing (i.e. linearising and normalizing, see below), these two photographs (visible and UV), yielded four image channels corresponding to the different parts of the spectrum, namely UV, short wavelength (SW), medium wavelength (MW), and long wavelength (LW) (see below). The sensitivity of the camera was previously calculated for the different wavelengths of light, while accounting for the filters and lens (see Stevens *et al.* (2014)^51^ and references therein). The sensitivities for each channel were: UV: 360–400 nm (peak 366 nm), SW: 400–550 nm (peak 465 nm), MW: 420–620 nm (peak 522 nm), LW: 560–700 nm (peak 667 nm).

The photography setup used for the experiments consisted of placing specimens on a 10 × 5 cm sheet of black ethylene-vinyl acetate (EVA), used as a low-UV reflective background (having less than 5% reflectance) (LMA, unpublished). Every photo contained a 40% Spectralon gray standard (Labsphere Congleton, UK) used for calibration. The ladybirds were photographed inside a darkroom on a camera stand to keep the distance from the lens and the individuals fixed at 60 cm. We used an EYE Color Arc ® MT70 bulb (Iwasaki Electric Co. Ltd) for illumination, which emits a spectrum similar to a D65 irradiance spectrum, enabling us to take images in UV wavelengths.

### Image calibration and analyses

In addition to determining the spectral sensitivity of our camera, we also corrected for the fact that most cameras have non-linear responses in image values to different light levels^14^ We therefore linearised each photograph using a set of Spectralon grey standards varying from 2 to 99% reflectance, as well as removing variation in light conditions with regards to the 40% reflectance standard and scaling the 8-bit images such that a pixel value of 255 equals 100% reflectance^14^. Calibrations were done using camera-specific custom plugins in Image J^52^.

Because we aimed to analyse the honesty of ladybird warning signals from a predator’s point of view, we used the reported cone sensitivities from the blue tit *Cyanistes caeruleus*^53^, to model avian visual perception, given that small birds are likely to be common predators of adult ladybirds^30^. We used these cone sensitivity values to transform our images to predicted cone catch values using a polynomial mapping technique with a D65 irradiance spectrum^14^. This technique is very effective and yields high R^2^ values (between 0.96–0.98) compared to cone catch values obtained through spectrometry^51^. Thus we obtained UV, SW, MW and LW and double cone mapped images, and measured the image values for each image channel for the background colour of the elytra (and plant samples) using the “Measure” command in Image J. The elytra of ladybird beetles is curved, thus we measured areas with no specular reflectance.

### Toxicity analyses

Here, we tested coccinellid toxicity using water fleas (*Daphnia pulex*) as a test organism since *Daphnia* have been shown to be excellent test subjects for ecotoxicology measures^54^. Additionally, they are readily available and sensitive to small amounts of chemicals dissolved in water. After taking the photographs, we weighed each ladybird to the nearest 0.01 g using a UMX2 ultra-microbalance; Mettler-Toledo Ltd., Leicester, UK. Each individual was stored in individual 1.5 ml centrifuge tubes and kept in a −20 ˚C freezer. Immediately before the toxicity assay, the elytra were removed using fine tweezers and stored in separate 1.5 ml centrifuge tubes. Carcasses were left to thaw for 20 min, and their weight recorded again. We weighed the ladybirds twice to determine if there was any loss of body weight after thawing the carcasses. After a preliminary test, we used the initial weight recorded for each individual. We added 0.5 ml Methanol 99% HPLC (Fisher Scientific, Inc) to the centrifuge tube to provide an aqueous phase for the toxins to dissolve. To extract the toxins in the ladybird’s haemolymph and body, each individual was macerated in its own centrifuge tube using a plastic conic pestle for two minutes. Each pestle was discarded afterwards to avoid any cross-contamination. After maceration, we centrifuged it at max G using an Eppendorff MiniSpin centrifuge for ten minutes. The supernatant was extracted into a 50 ml glass test tube using a 200 μl Gilson micropipette, and the pellet and centrifuge tubes discarded. To obtain the extracted toxins we evaporated the excess methanol using a Thermo Savant ISS110 SpeedVac concentrator set at high temperature for 35 min. This procedure ensured that the toxic effects of the methanol would not affect the results of the tests. Once the methanol was evaporated, we added 1.75 ml ultrapure water (Milli-Q Synthesis; Millipore, Watford, UK) and using a vortex, we homogenised the sample again. This new solution was used as our stock extract. From the stock, we took 7 separate aliquots corresponding to 100%, 80%, 60%, 50%, 40%, 20% and 0%, toxin and added them to 15 ml Falcon vials completing the volume up to 0.5 ml on every dilution (except the 100%) with ultra pure water. We repeated this procedure using (1) only ultra pure water and (2) only methanol solutions, to have controls of the experimental procedure respectively and to ensure that methanol itself was not highly toxic.

The toxicity of the extracts and dilutions described above was tested on adult water fleas, *Daphnia pulex* obtained from Sciento Biological Supplies (Manchester, UK). We added ten *D. pulex* to each vial and counted the number of dead individuals after 1 (one), 3 (three), and 24 hours. The vials were maintained in a constant-environment room 19.7 ˚C, 61% relative humidity and 24 h light regimen.

### Signal honesty and correlations between colour and toxicity

We aimed to determine the relationship between several coloration attributes and ladybird toxicity to be able to establish if ladybird colours are an honest indicator of ladybird defenses. For each species we measured five attributes of coloration that reflect both the absolute properties of individuals, and their conspicuousness against the background. These aspects were: 1) Luminance, which refers to the overall achromatic information or intensity of an object. This measure was given by the double cone value of the image since evidence indicates that achromatic vision in birds is mediated by the double cones^29^. 2) Saturation can be defined as the perceived intensity of a colour, or how much of a colour type there is compared to white light (e.g. red versus pink). We measured saturation by determining the Euclidian distance of each colour from the achromatic center of a cone-sensitivity weighted tetrahedral colour space. 3) We measured the area of the background colour using a centimeter scale in each of the photos and using the polygon selection tool on Image J. 4) In addition, we calculated the colour and luminance contrasts of each ladybird species against the specific plant that we found them on upon collection. For these measurement we used a log form of the Vorobyev – Osorio model^55^. The model takes into account the sensitivity of each cone in the predator’s visual system and estimates of cone abundance and noise in the photoreceptors to calculate discrimination thresholds or ‘just noticeable differences’ (JND), whereby values close to 1.00–3.0 indicate that two objects are likely to be indistinguishable to an observer (avian), and values above this are increasingly likely to lead to detection and discrimination^55^. Colour contrast was calculated using the LW, MW, SW and UV values obtained from the photo while the luminance contrast uses the double cone values. 5) Finally, we measured the contrast of the background colour of the elytra against the spot colours, as a measure of “internal contrast”. For these measures we used the model described above. However, we only used the luminance values for this calculation. This is because comparing a chromatic background (red, orange) with an achromatic spot (black, white) or *vice versa* may not be biologically relevant.

### Phylogenetic comparative analyses

We used the phylogeny obtained by Magro *et al.* (2010)^56^ to determine if the coloration and toxicity of ladybirds had any association with their relatedness. However, since this study does not include the Larch ladybird, we reconstructed the relationships of our species using additional sequences from Genbank (http://www.ncbi.nlm.nih.gov/genbank/); accession numbers: HM909101 and KJ963033. We built three different phylogenies using Neighbour Joinig, Maximum parsimony and RaxML criteria using the R packages “ape”, “phangorn” and “phyloch”. Because our reconstruction yielded unresolved polytomies we calculated both the K (Blomberg *et al.* (2003)^57^ and λ (Pagel (1999)^58^) estimators for phylogenetic signal for all the possible phylogenies of our species (see Supplementary material S1). However, these parameters were non-significant for both of our main predicting variables, suggesting no role of phylogeny in our results. Note, however, that this lack of association should be interpreted with caution because of the small number of independent replicates in the phylogeny that may not have enough resolution to reveal true associations^28^. Overall, given the lack of evidence for phylogenetic effects here, coupled with previous recent evidence that there is no influence of phylogeny on coloration or toxicity when using similar methods^6^, we continued our analyses without further controls for phyogeny.

### Artificial ladybird models

We aimed to measure predation rates on prey models resembling the different ladybird species in the environment. For this purpose, we made artificial ladybird models that resembled, to predator (avian) vision, the colours of our five species. To match the colours of the ladybirds we used the individuals that we captured for the toxicity tests to calculate the average avian cone catch values on each wavelength for each species (see above). Using these average values as references, a grid of similar colours to each of the species was generated using the CYMK scale on Adobe Photoshop CS. We printed the grids on white paper (Navigator High Quality A4 Presentation Paper 100 gsm) using an HP LaserJet enterprise M551DN printer. We covered the chosen colour with a double layer of non-toxic Pritt Craft PVA glue, to make the models stronger, weather-resistant and shiny to simulate the real appearance of ladybirds. Once printed and covered with glue, we used the same methods described in Merrill *et al.* 2012^23^ to determine which artificial colour matched the real ladybirds best. This method ensured that the colours that we matched to the real ladybirds took into account the influence of the layer of coating when calculating the reflectance values of the models. Our previous data shows that all ladybird species here have very low UV reflectance^8^. Furthermore, Lyytinen (2001)^59^ found that ultraviolet signal components are unlikely to play an important role in aposematism. Thus, here we designed the ladybird prey with regards to the visible spectrum of ladybird signals. This includes only information between 400 and 700 nm. Note, however, that our analysis of ladybird coloration and conspicuousness against the background (both real ladybirds and models) did consider UV information. This is important because even though ladybirds reflect low UV light, natural backgrounds may have UV reflectance, thereby generating contrast in the UV part of the spectrum.

Once the colours of the models were chosen and coated, we proceeded to make the ladybird shaped paper models. We used the average size of the all the ladybirds (5 mm) collected for the toxicity tests as a proxy to choose the size of the artificial models (see *study species and sites* section). We built a positive and negative Polymethyl methacrylate (PMMA) acrylic mold using a CNC milling machine (constructed by Joylon Troscianko, University of Exeter). The printed paper was shaped using the mold, and filled with Blu-Tack pressure-sensitive adhesive for the model to hold its shape. To ensure that the models would not be blown away, we put an extra layer of glue on the inside of the model, in between the paper and the BluTack.

### Survival experiment

We used a randomized block design to determine the different predation rates of the ladybird species in this study. We placed our models along 15 randomly selected 250 m transects along the Southwest Coastal Paths between Falmouth, Cornwall (50° 8′33.16′′N, 5° 4′13.70′′W) and Maenporth, Cornwall (50°′7′6.916′′N, 5°′5′25.9146′′W). The models were placed at heights between 0.2 and 1.2 m every 5 m using a drawing pin to hold them to a leaf. The pin also provided extra support for each model. We chose species of plants where ladybirds are usually found basking to place our models. The species of plants were: common nettles (*Urtica dioica*), blackberry bushes (*Rubus fruticosa),* pink campion (*Silene dioica*), hogweed (*Heracleum sphondylium*), sorrel (*Rumex acetosa*), English ivy (*Hedera helix),* foxglove *(Digitalis purpurea)* and fairy foxglove *(Erinus alpinus)*.

We determined that the checking times should be every 4 hours over a total of 48 h exposure to potential avian predators. The models were considered to be attacked if they could not be found on the leaf or if there were clear peck marks^23^. We considered the models to be censored if there were small bite marks probably from a rodent, where normal degradation of the model occurred, or if there was a slime trail present (evidence of a snail or slug). A preliminary analysis showed that the survival curves were similar with and without censored data (ANOVA, DF = 1; F = 82.66; p = 0.190). Thus we here present the results without any ambiguous predation cases. A preliminary survey on three transects showed that we could easily determine when the models had been attacked as opposed to being degraded (see Supplementary material S5).

In addition, to determine the variation in contrast of the models against natural backgrounds we took calibrated photographs of 15 randomly chosen models in each transect. These photographs were taken with the same camera specifications described above, and the contrast of each photographed model was calculated using the same method described in the section *“Signal honesty and correlations between colour and toxicity”*.

### Statistical analyses

All our analyses were made using R version 3.1.0 “Spring Dance”^60^. In order to determine the differences in lethality of the ladybird species used, we performed a survival analysis using the package ‘survival’ version 2.37–7. We analysed the survival curves using a Cox Mixed Effects model. For each toxin dilution and each species we performed a survival pairwise comparison using the fixed coefficients of the survival analysis (included in the R-package). This approach was also used to determine the probability of survival of the artificial models.

To establish whether ladybird warning colours are an honest indicator of toxicity we performed a Linear Mixed Effect model. We used the average number of dead Daphnia per species after three (3) hours as an indicator of toxicity. This time point was chosen because the information of the first check after one hour may have been too early to determine the real effects of the toxins, whereas the information after 24 hours could have been affected by the *Daphnia* not having food for a longer period of time and could also miss variation in toxicity among species/individuals if most *Daphnia* had died by this point. In any case, a simple repeated measures analysis showed that the three time periods used to assess *Daphnia* mortality were indistinguishable (ANOVA (RM), Species*hour, DF = 5, 14, F = 0.254, p = 0.997). The distribution of the three hour data was non-normal, and so we performed a Log-transformation on this data (Kolmogorov-Smirnoff, p = 0.08; Shapiro-Wilk, p = 0.06, after transformation). In addition, we calculated the lethal concentration for each species to kill 50% of the Daphnia in each concentration (LC50). This is a common measure in ecotoxicology tests^54^. However, we aimed to measure the inter-individual variations in signal honesty and used a limited number of toxin concentrations in our analyses. Thus, we did not take into account the LC50 results to make predictions about signal honesty. Supplementary material S6 shows a graph of each species’ LC50, which concurs with the degree of toxicity analysed through the number of dead Daphnia after 3 hours.

As predictive variables for the toxicity of the ladybirds, we included the fixed effects of species, size (length), weight, luminance, saturation, area of background coloration of the elytra, contrast against each species’ own background, each species’ internal contrast (in luminance values). In addition, we included each ladybird’s ID as a random factor and included each possible two-way interaction in the model. We also controlled for the weight (initial), and length of each species, as these might have an effect on the amount of toxin that each individual had. We examined the dispersion of our data and the residuals using the LMER Convenience Functions package. The residual plots showed a good distribution of the residuals. The model was simplified by dropping any non significant terms using the fitLMER.fnc relLik.AIC functions. Markov Chain Monte Carlo (MCMC) post-hoc comparisons on the relevant variables where carried out using the mcposthoc.fnc function.

To determine whether the artificial model survival was different, we also used a Cox Mixed Effects model. Further to correlate survival effects to characteristics of coloration, we used a Linear Mixed Effect model, with the block and individual ID as random factors. The dispersion of the data was determined using the LMER Convenience Functions package, which showed a distribution of the residual errors within +/−2SD. After running the model, MCMC post –hoc comparisons between each colour (species) were made using the mcposthoc.fnc function.

## Additional Information

**How to cite this article**: Arenas, L.M. *et al.* Signal honesty and predation risk among a closely related group of aposematic species. *Sci. Rep.* **5**, 11021; doi: 10.1038/srep11021 (2015).

## Supplementary Material

Supplementary Information
