# Supplementary material for: Signal honesty and predation risk among a closely related group of aposematic species
Source: Sci Rep. 2015 Jun 5;5:11021. doi: 10.1038/srep11021 (PMC4457162; doi:10.1038/srep11021)
Supplement: Supplementary Information [file srep11021-s1.pdf]

Signal honesty and predation risk among a closely related group of aposematic species – Supplementary Information

Lina María Arenas*[1,2], Dominic Walter[2] and Martin Stevens [2]

1. Department of Zoology, University of Cambridge, Downing Street, CB2 3EJ, UK.

2. Centre for Ecology & Conservation, College of Life & Environmental Sciences, University of Exeter, Penryn Campus, Penryn, Cornwall, TR10 9FE, UK.

*Author for correspondence; email: lma38@cam.ac.uk

Supplementary material S1:

Sequence acquisition and alignment

To test for possible influences of phylogeny on our results, we downloaded the same sequences used by by Magro et al. (2010) for each of our species from Genbank (http://www.ncbi.nlm.nih.gov/ genbank/). In addition, we downloaded two sequences for the Larch ladybird given that this species has not been included in previous studies (accession numbers: HM909101 and KJ963033). We aligned the sequences using CLUSTALW (Thompson et al., 1994), and adjusted this alignment by eye.

Phylogenetic reconstruction

We assessed the phylogenetic relationship of our five species using three criteria: Neighbour Joining (NJ), Maximum Parsimony (MP), and Maximum likelihood (ML). The phylogenies were reconstructed in R using the 'ape', 'phangorn' and 'phyloch' packages. For the ML approach, we used RAxML version 7.0.4 (Stamatakis et al., 2005) with default settings (GTRGAMMA model). These three reconstruction yielded unresolved polytomies. Thus we used the 'resolve.root' command to build five (5) trees for each criteria forcing the root to each of our five species, for a total of 15 plausible phylogenetic reconstructions. Using the package and 'picante' we calculated the K estimator for phylogenetic signal (Blomberg et al. 2003) for our main predicting variables, namely, Contrast against the background and Saturation, for every possible reconstruction. These calculations yielded high values of K but with non-significant p values (e.g. Contrast: K=0.91; p=0.43; Saturation: K= 0.85; p=0.44) for all of our possible reconstructions. Furthermore, using the package 'phytools' we calculated the estimator λ (Pagel 1999), which yielded similar results, with very low λ, but non-significant p values (e.g. Contrast: λ= 0.00; p=1.00; Saturation: λ =0.00; p=1.00) for

all topologies.

Supplementary material 2: Survival analysis pairwise comparisons based on the fixed coefficients of each toxin dilution and species

Species/Dilution	Orange ladybird	2-spot (typica)	2-spot (melanic)	Pine ladybird	14-spot ladybird	Larch ladybird	H2O control	MeOH Control	100
Orange ladybird									
2-spot (typica)	**coef= 0.04; z= -0.61; p=0.54**								
2-spot (melanic)	**coef= 0.09; z= 1.23; p=0.22**	**coef= 0.14; z= 1.87; p=0.06**							
Pine ladybird	coef= -0.27; z= -3.51; p<0.000	coef= -0.22; z= -2.96; p<0.000	coef= -0.36; z= -4.82; p<0.000						
14-spot ladybird	coef= -0.28; z= -3.57; p<0.000	coef= -0.23; z= -3.02; p<0.000	coef= -0.37; z= -4.84; p<0.000	**coef= 0.01; z= 0.13; p=0.9**					
Larch ladybird	coef= -0.93; z= -11.24; p<0.000	coef= -0.88; z= -10.74; p<0.000	coef= -1.02; z= -12.40; p<0.000	coef= -0.65; z= -7.95; p<0.000	coef= -0.64; z= -7.71; p<0.000				
H2O control	coef= -2.95; z= -13.35; p<0.000	coef= -2.90; z= -13.15; p<0.000	coef= -3.05; z= -13.79; p<0.000	coef= -2.68; z= -12.13; p<0.000	coef= -2.67; z= -12.04; p<0.000	coef= -2.02; z= -9.07; p<0.000			
MeOH Control	coef= -1.50; z= -11.17; p<0.000	coef= -1.46; z= -10.85; p<0.000	coef= -1.60; z= -11.90; p<0.000	coef= -1.23; z= -9.18; p<0.000	coef= -1.22; z= -9.01; p<0.000	coef= -0.57; z= -4.19; p<0.000	coef= 1.44; z= 5.89; p<0.000		

Species/Dilution	Orange ladybird	2-spot (typica)	2-spot (melanic)	Pine ladybird	14-spot ladybird	Larch ladybird	H2O control	MeOH Control	80
Orange ladybird									
2-spot (typica)	coef= -0.24; z= -3.20; p<0.000								
2-spot (melanic)	**coef= 0.03; z= -0.45; p=0.65**	coef= -0.21; z= 2.81; p<0.000							
Pine ladybird	coef= -0.50; z= -6.39; p<0.000	coef= -0.25; z= -3.28; p<0.000	coef= -0.46; z= -6.07; p<0.000						
14-spot ladybird	coef= -0.58; z= -7.32; p<0.000	coef= -0.33; z= -4.27; p<0.000	coef= -0.55; z= -7.00; p<0.000	**coef= -0.08; z= -1.06; p=0.29**					
Larch ladybird	coef= -1.18; z= -14.06; p<0.000	coef= -0.93; z= -11.14; p<0.000	coef= -1.14; z= -13.68; p<0.000	coef= -0.68; z= -8.04; p<0.000	coef= -0.59; z= -6.93; p<0.000				
H2O control	coef= -2.69; z= -14.03; p<0.000	coef= -2.44; z= -12.74; p<0.000	coef= -2.65; z= -13.87; p<0.000	coef= -2.19; z= -11.42; p<0.000	coef= -2.10; z= -10.92; p<0.000	coef= -1.51; z= -7.77; p<0.000			
MeOH Control	coef= -1.81; z= -12.79; p<0.000	coef= -1.56; z= -11.05; p<0.000	coef= -1.78; z= -12.57; p<0.000	coef= -1.31; z= -9.26; p<0.000	coef= -1.22; z= -8.58; p<0.000	coef= -0.63; z= -4.36; p<0.000	coef= 0.87 z= 3.92; p<0.000		

Species/Dilution	Orange ladybird	2-spot (typica)	2-spot (melanic)	Pine ladybird	14-spot ladybird	Larch ladybird	H2O control	MeOH Control	60
Orange ladybird									
2-spot (typica)	coef= -0.20; z= -2.59; p<0.000								
2-spot (melanic)	**coef= 0.16; z= 2.08; p=0.02**	**coef= 0.03; z= 0.40; p=0.69**							
Pine ladybird	coef= -0.55; z= -7.00; p<0.000	coef= -0.35; z= -4.53; p<0.000	coef= -0.38; z= -4.89; p<0.000						
14-spot ladybird	coef= -0.74; z= -9.10; p<0.000	coef= -0.54; z= -6.73; p<0.000	coef= -0.57; z= -7.06; p<0.000	coef= 0.19; z= -2.33; p<0.05					
Larch ladybird	coef= -1.52; z= -16.71; p<0.000	coef= -1.32; z= -14.56; p<0.000	coef= -1.35; z= -14.76; p<0.000	coef= -0.97; z= -10.56; p<0.000	coef= -0.78; z= -8.29; p<0.000				
H2O control	coef= -2.65; z= -13.20; p<0.000	coef= -2.45; z= -12.21; p<0.000	coef= -2.48; z= -12.36; p<0.000	coef= -2.10; z= -10.44; p<0.000	coef= -1.91; z= -9.43; p<0.000	coef= -1.12; z= -5.47; p<0.000			
MeOH Control	coef= -1.81; z= -12.04; p<0.000	coef= -1.61; z= -10.73; p<0.000	coef= -1.64; z= -10.92; p<0.000	coef= -1.26; z= -8.37; p<0.000	coef= -1.07; z= -7.01; p<0.000	coef= -0.28; z= -1.83; p<0.000	coef= 0.84; z= 3.54; p<0.000		

Species/Dilution	Orange ladybird	2-spot (typica)	2-spot (melanic)	Pine ladybird	14-spot ladybird	Larch ladybird	H2O control	MeOH Control	50
Orange ladybird									
2-spot (typica)	coef= -0.38; z= -4.87; p<0.000								
2-spot (melanic)	coef= -0.21; z= -2.77; p<0.000	**coef= 0.16; z= 2.08; p=0.03**							
Pine ladybird	coef= -0.52; z= -6.55; p<0.000	**coef= 0.13; z= 1.75; p=0.08**	coef= -0.30; z= -3.79; p<0.000						
14-spot ladybird	coef= -1.82; z= -11.85; p<0.000	coef= -0.64; z= -7.41; p<0.000	coef= -0.80; z= -9.27; p<0.000	coef= -0.50; z= -5.77; p<0.000					
Larch ladybird	coef= -1.47; z= -16.11; p<0.000	coef= -1.09; z= -11.84; p<0.000	coef= -1.26; z= -13.55; p<0.000	coef= -0.95; z= -10.29; p<0.000	coef= -0.45; z= -4.58; p<0.000				
H2O control	coef= -2.74; z= -12.39; p<0.000	coef= -2.35; z= -10.64; p<0.000	coef= -2.52; z= -11.38; p<0.000	coef= -2.21; z= -10.01; p<0.000	coef= -1.71; z= -7.64; p<0.000	coef= -1.26; z= -5.58; p<0.000			
MeOH Control	coef= -2.30; z= -12.37; p<0.000	coef= -1.92; z= -10.28; p<0.000	coef= -2.08; z= -11.16; p<0.000	coef= -1.78; z= -9.54; p<0.000	coef= -1.27; z= -6.73; p<0.000	coef= -0.82; z= -4.30; p<0.000	**coef= 0.43; z= 1.57; p=0.12**		

Species/Dilution	Orange ladybird	2-spot (typica)	2-spot (melanic)	Pine ladybird	14-spot ladybird	Larch ladybird	H2O control	MeOH Control	40
Orange ladybird									
2-spot (typica)	coef= -0.41; z= -5.05; p<0.000								
2-spot (melanic)	coef= -0.28; z= -3.46; p<0.000	**coef= 0.12; z= 1.53; p=0.13**							
Pine ladybird	coef= -0.55; z= -6.74; p<0.000	**coef= -0.14; z= -1.74; p=0.08**	coef= -0.27; z= -3.24; p<0.000						
14-spot ladybird	coef= -1.16; z= -12.59; p<0.000	coef= -0.75; z= -8.11; p<0.000	coef= -0.88; z= -9.41; p<0.000	coef= -0.60; z= -6.52; p<0.000					
Larch ladybird	coef= -1.55; z= -15.66; p<0.000	coef= -1.13; z= -11.40; p<0.000	coef= -1.26; z= -12.56; p<0.000	coef= -0.99; z= -9.91; p<0.000	coef= -0.38; z= -3.53; p<0.000				
H2O control	coef= -2.93; z= -10.95; p<0.000	coef= -1.91; z= -8.99; p<0.000	coef= -2.04; z= -9.57; p<0.000	coef= -1.77; z= -8.31; p<0.000	coef= -1.16; z= -5.34; p<0.000	coef= -0.78; z= -3.54; p<0.000			
MeOH Control	coef= -2.35; z= -11.05; p<0.000	coef= -1.93; z= -9.09; p<0.000	coef= -2.06; z= -9.67; p<0.000	coef= -1.79; z= -8.41; p<0.000	coef= -1.18; z= -5.44; p<0.000	coef= -0.80; z= -3.64; p<0.000	**coef= -0.22; z= -0.08; p=0.94**		

Supplementary material 2: Survival analysis pairwise comparisons based on the fixed coefficients of each toxin dilution and species

**Species/Dilution**	**Orange ladybird**	**2-spot (typica)**	**2-spot (melanic)**	**Pine ladybird**	**14-spot ladybird**	**Larch ladybird**	**H2O control**	**MeOH Control**	**20**
Orange ladybird									
2-spot (typica)	coef= -0.29; z= -3.14; p<0.000								
2-spot (melanic)	**coef= -0.09; z= -1.04; p=0.30**	**coef= 0.19; z= 2.10; p=0.36**							
Pine ladybird	coef= -0.27; z= -2.85;p<0.000	**coef= 0.02; z= 0.24; p=0.81**	**coef= -0.17; z= -1.82; p=0.06**						
14-spot ladybird	coef= -0.67; z= -6.47; p<0.000	coef= -0.37; z= -3.60; p<0.000	coef= -0.57; z= -5.53; p<0.000	coef= -0.40; z= -3.77; p<0.000					
Larch ladybird	coef= -1.17; z= -9.92; p<0.000	coef= -0.87; z= -7.34; p<0.000	coef= -1.07; z= -9.04; p<0.000	coef= -0.89; z= -7.47; p<0.000	coef= -0.49; z= -3.42; p<0.000				
H2O control	coef= -1.42; z= -6.85; p<0.000	coef= -1.12; z= -5.40; p<0.000	coef= -1.32; z= -6.37; p<0.000	coef= -1.14; z= -5.50; p<0.000	coef= -0.74; z= -3.51; p<0.000	**coef= -0.25; z= -1.14; p=0.25**			
MeOH Control	coef= -1.52; z= -6.94; p<0.000	coef= -1.22; z= -5.57 p<0.000	coef= -1.42; z= -6.49; p<0.000	coef= -0.25; z= -5.66; p<0.000	coef= -0.48; z= -3.78; p<0.000	**coef= -0.35; z= -1.52; p=0.13**	**coef= -0.10; z= -0.35; p=0.72**		

**Species/Dilution**	**Orange ladybird**	**2-spot (typica)**	**2-spot (melanic)**	**Pine ladybird**	**14-spot ladybird**	**Larch ladybird**	**H2O control**	**MeOH Control**	**0**
Orange ladybird									
2-spot (typica)	**coef= -0.15; z= -1.07; p=0.29**								
2-spot (melanic)	**coef= 0.01; z= 0.12; p=0.91**	**coef= 0.17; z= 1.18; p=0.24**							
Pine ladybird	**coef= -0.26; z= -1.73; p=0.84**	**coef= -0.10; z= -0.72; p=0.47**	**coef= 0.17; z= 1.18; p=0.06**						
14-spot ladybird	**coef= -0.16; z= -1.07; p=0.29**	**coef= -0.006; z= -0.04; p=0.97**	**coef= -0.27; z= -1.84; p=0.24**	**coef= 0.10; z= 0.65; p=0.51**					
Larch ladybird	**coef= -0.11; z= -0.78; p=0.44**	**coef= 0.03; z= 0.26; p=0.79**	**coef= -0.13; z= -0.89; p=0.37**	**coef= 0.14; z= 0.95; p=0.34**	**coef= 0.04; z= 0,29; p=0.77**				
H2O control	**coef= -0.06; z= -0.29; p=0.77**	**coef= 0.09; z= 0.44; p=0.66**	**coef= -0.07; z= -0.37; p=0.71**	**coef= 0.20; z= 0.92; p=0.36**	**coef= 0.09; z= 0.46; p=0.65**	**coef= 0.05; z= 0.25; p=0.8**			
MeOH Control	**coef= 0.13; z= 0.68; p=0.49**	**coef= 0.29; z= 1.45; p=0.15**	**coef= 0.12; z= 0.60; p=0.55**	**coef= 0.39; z= 1.95; p=0.05**	**coef= 0.29; z= 1.45; p=0.15**	**coef= 0.25; z= 1.24; p=0.21**	**coef= 0.19; z= 0.79; p=0.43**		

Supplementary figure 3: The figure shows the measurements for the internal contrast of the ladybird elytra. These measurements are based on luminance values, as comparing chromatic (red, orange) against achromatic (black, white) is not a reliable method.

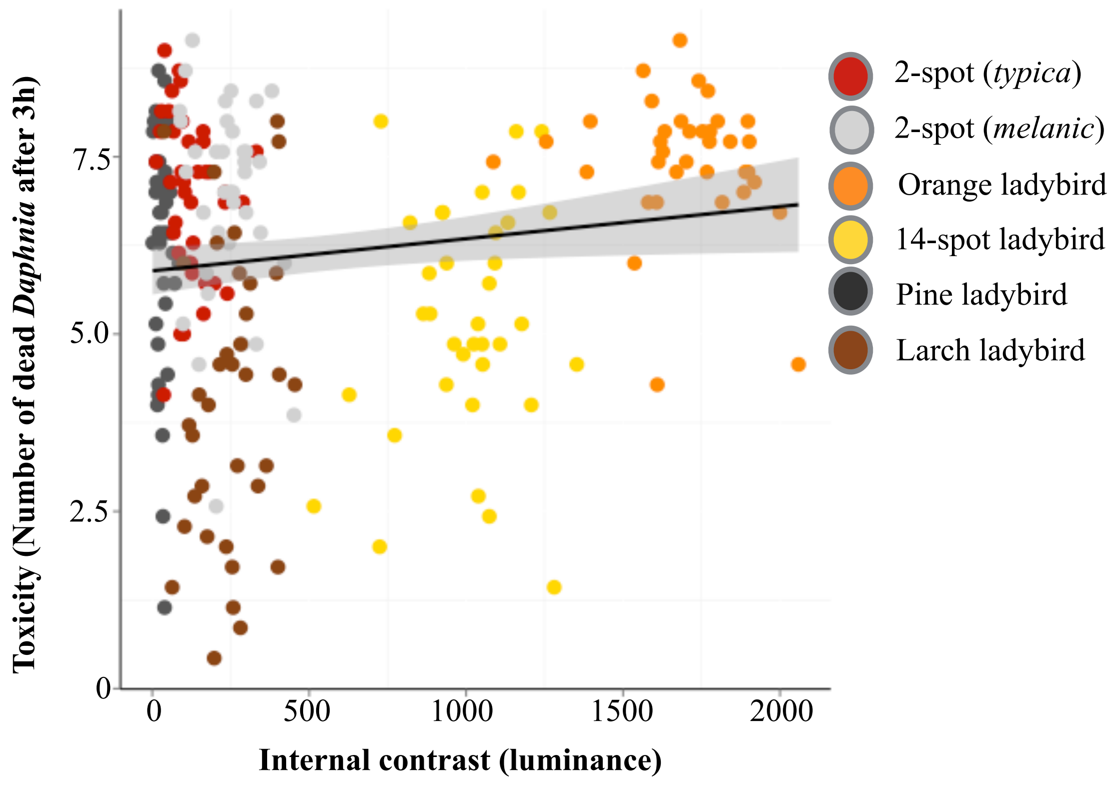

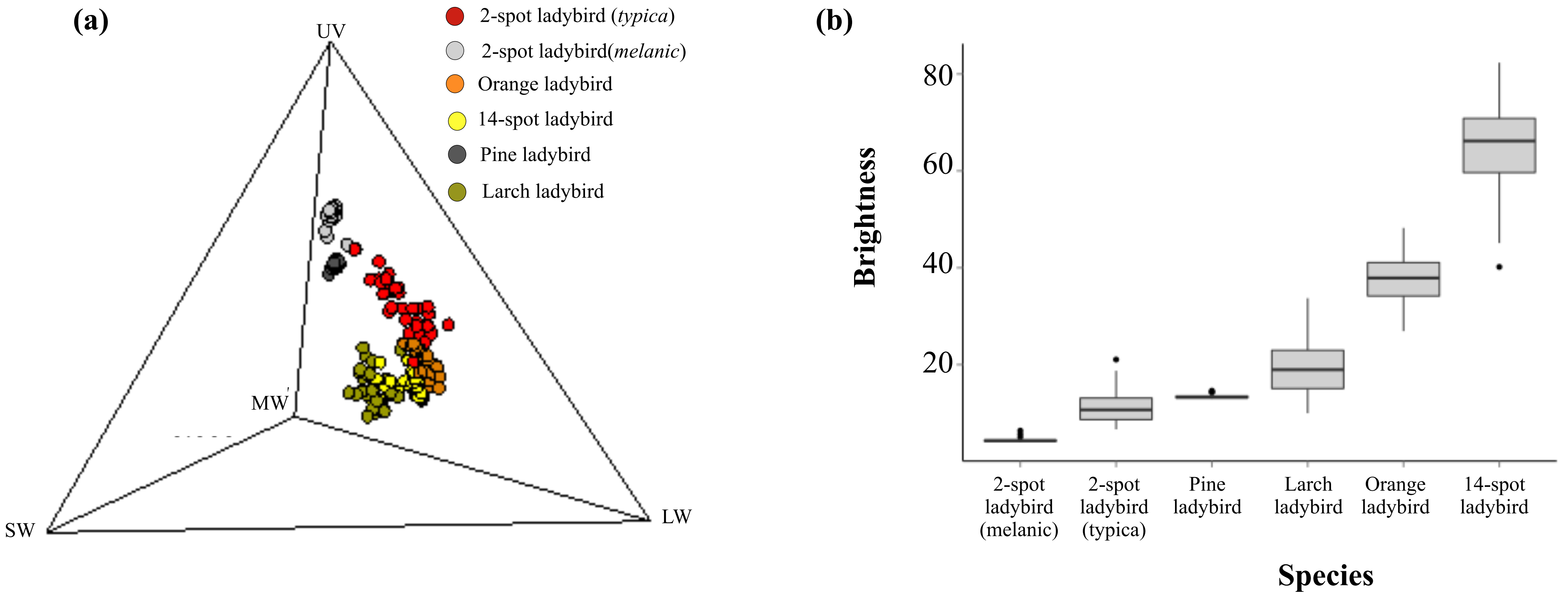

Supplementary Figure 4: (a) Representation of each species of ladybird in a four - dimensional colour space. The axes on the tetrahedron have been weighted according to the cone sensitivity of a passerine bird. (b) The figure shows the brightness calculations for each species included in this study.

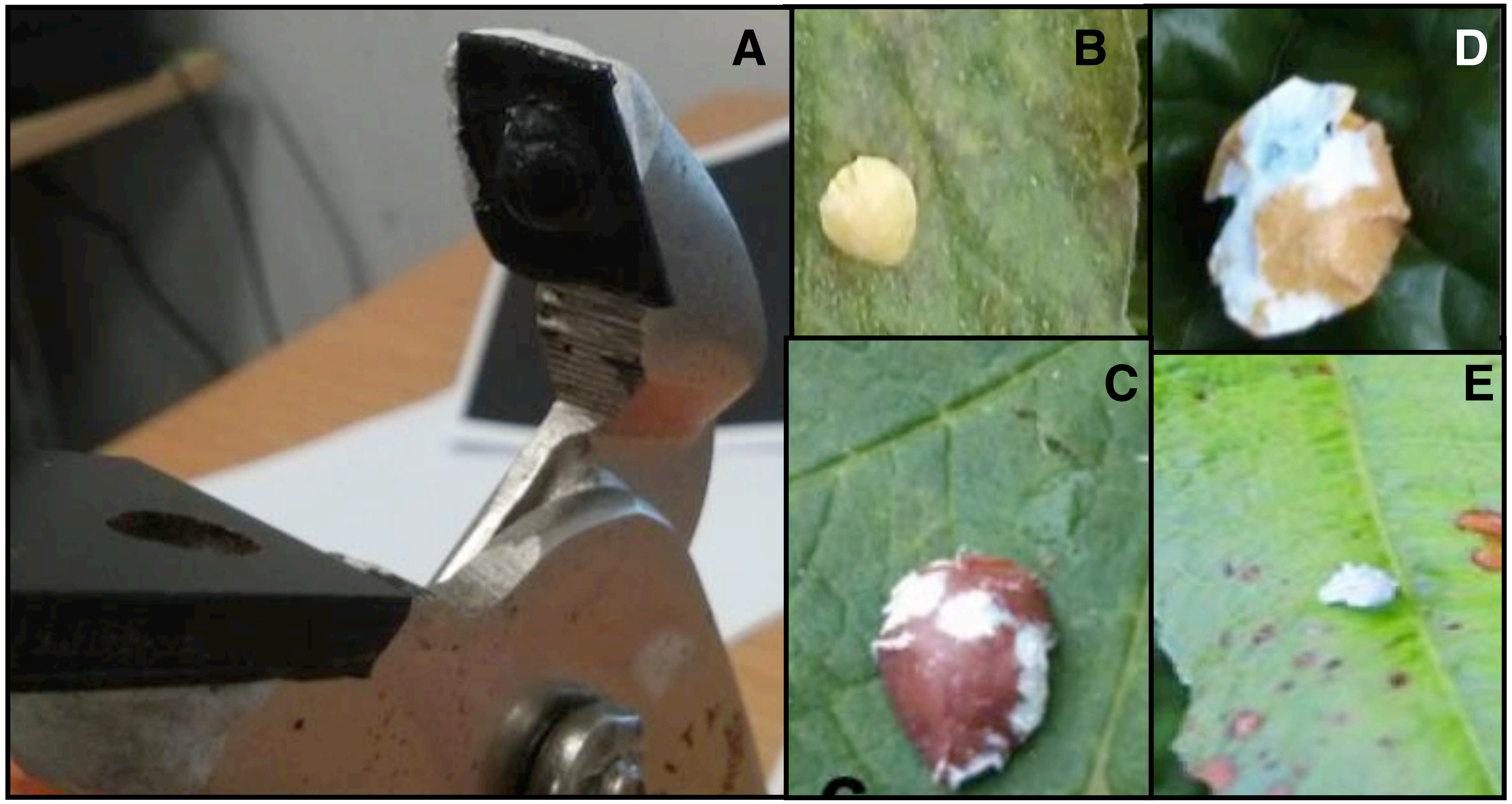

Supplementary figure 5: The figure shows the paper models used for the predation risk experiments carried out in this study. Figure 4A shows the acrylic positive-and negative mold designed to model the ladybirds to an average size between the species collected for the toxicity tests. Figure 4B shows the model pinned to a leaf using Blue-Tack adhesive and a drawing pin to hold it to the leaf. Figures 4C, 4D, and 4E shows the types of attacks we quantified in our experiments. These ranged from the model being pecked or bitten to the complete consumption of the model.

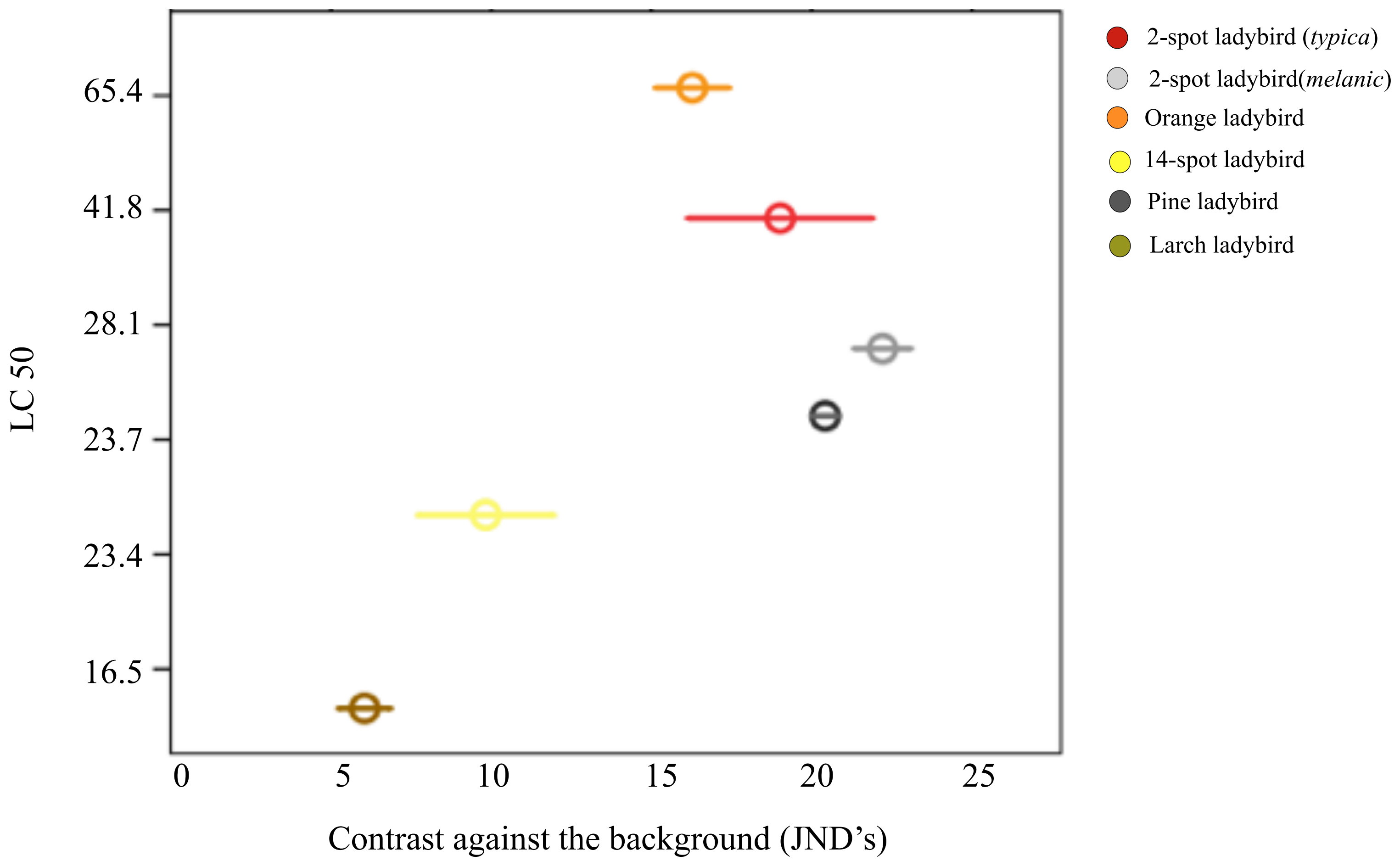

Supplementary figure 6: The figure shows the relationship between the measures of Lethal Concentration 50 (LC50) and the contrast against the background (measured in JNDs) for the five species used in this study. These results yielded the same tendencies as the results presented with the number of Daphnia dead after 3 hours.
